# Genetic factors in the development of suicidal ideation – results of a GWAS study

**DOI:** 10.1192/j.eurpsy.2025.513

**Published:** 2025-08-26

**Authors:** B. Erdelyi-Hamza, D. Torok, Z. Gal, D. Gyorik, N. Eszlari, S. Krause, G. Bagdy, G. Juhasz, X. Gonda

**Affiliations:** 1 Department of Psychiatry and Psychotherapy; 2 Doctoral School of Mental Health Sciences; 3 Faculty of Pharmacy, Department of Pharmacodynamics; 4 NAP3.0 Neuropsychopharmacology Research Group, Hungarian Brain Research Program, Semmelweis University, Budapest, Hungary

## Abstract

**Introduction:**

Psychiatric disorders are risk factors for suicidality, but it is also considered a multifactorial phenomenon. Genome-wide association studies may provide an insight into the etiological background of distinct phenomena along the suicidal continuum, and may also help disentangle their overlapping genetic architecture with psychiatric disorders.

**Objectives:**

Our aim was to carried out a GWAS for suicidal ideation in a well-phenotyped sample.

**Methods:**

We conducted a genome-wide association study (GWAS) in the NewMood (New Molecules in Mood Disorders, Manchester and Budapest) database, 1820 subjects were involved (533 males, 1287 females), the suicidal ideation and behaviour were investigated with a suicide-focused item of the Brief Symptom Inventory (BSI). SNP-level association was assessed employing linear regression models, assuming additive genetic effects, using PLINK2.0, with gender, age and the first ten principal components (PCs) of the genetic data as covariates. Bonferroni-corrected significance threshold on SNP-level was p ≤ 5.0 × 10-8, and the suggestive significance threshold was p ≤ 1.0 × 10-5. GWAS results including the identified significant results were interpreted using FUMA v1.5.2.

**Results:**

9 SNPs were identified, 2 with genome-wide significance and 7 with suggestive significance. The most significant SNP, *rs79912020* (β=, P=3.21x10^-10^, Chr4) was located in the *MANBA* gene and the other genome-wide significant variant, rs10236520 (β=, P= 1.706x10^-8^, Chr7) is located near the gene *LOC124901613.* Furthermore, we have found more important variants with suggestive significance, rs117677616 (β= , P=1.199x10^-6^, Chr20) is identified in *PTPRT* gene, rs34475 (β= , P=1.981x10^-6^, Chr12) is located in *CFAP54*, rs711180 (β= , P= 2.934x10^-6^, Chr12) is near the gene *VWA1* and the variant rs2655484 (β= , P=5.717x10^-6^, Chr12) is located near the *GRIP1.* No genes were identified in gene-level analysis with genome-wide significance.

**Conclusions:**

We identified 9 SNPs with genome-wide or suggestive significance in association with suicidal ideation, with several lines of converging evidence supporting their involvement in the development of suicide risk. *MANBA* gene has role in the development of unipolar depression, *PTPRT* is associated with appearance of major depressive disorder, and the *GRIP1* gene may be considered also as a potential biomarker for suicide, as it has been previously associated with psychiatric phenotypes indirectly linked to suicidal behaviour and in patients with increasing suicidal ideation during antidepressant treatment. The prevention of the suicides is a prominent aim in mental healthcare and these new variants may be helpful in establishing novel, focused and more filters for this vulnerability.

Funding: NAP2022-I-4/2022, K143391, 2019-2.1.7-ERA-NET-2020-00005, TKP2021-EGA-25

**Disclosure of Interest:**

None Declared

